# Presenilin-1 Mutations Are a Cause of Primary Lateral Sclerosis-Like Syndrome

**DOI:** 10.3389/fnmol.2021.721047

**Published:** 2021-08-30

**Authors:** Juan Francisco Vázquez-Costa, María Payá-Montes, Marina Martínez-Molina, Teresa Jaijo, Jazek Szymanski, Miguel Mazón, Pablo Sopena-Novales, ENoD Consortium, Beatriz Morte, Jordi Pérez-Tur, Teresa Sevilla

**Affiliations:** ^1^Neuromuscular Unit and ERN-NMD Group, Department of Neurology, Hospital Universitario y Politécnico La Fe, Valencia, Spain; ^2^Centro de Investigación Biomédica en Red de Enfermedades Raras, Valencia, Spain; ^3^Department of Medicine, University of Valencia, Valencia, Spain; ^4^Genetics Department, Hospital Universitario y Politécnico La Fe, Valencia, Spain; ^5^Molecular Genetics Unit, Institut de Biomedicina de València-CSIC, Valencia, Spain; ^6^Centro de Investigación Biomédica en Red de Enfermedades Neurodegenerativas, Valencia, Spain; ^7^Department of Radiology and Biomedical Imaging Research Group GIBI230, Hospital Universitario y Politécnico La Fe and Instituto de Investigación Sanitaria La Fe, Valencia, Spain; ^8^Nuclear Medicine Department, Hospital Universitario y Politécnico La Fe, Valencia, Spain; ^9^Mixed Unit of Neurology and Genetics, Instituto de Investigación Sanitaria La Fe, València, Spain

**Keywords:** primary lateral sclerosis, progressive spastic paraparesis, Alzheimer’s disease, *PSEN1* mutation, motor neuron disease

## Abstract

**Background and Purpose:**

Primary lateral sclerosis (PLS) is a progressive upper motor neuron (UMN) disorder. It is debated whether PLS is part of the amyotrophic lateral sclerosis (ALS) spectrum, or a syndrome encompassing different neurodegenerative diseases. Recently, new diagnostic criteria for PLS have been proposed. We describe four patients of two pedigrees, meeting definite PLS criteria and harboring two different mutations in presenilin 1 (*PSEN1*).

**Methods:**

Patients underwent neurological and neuropsychological examination, MRI, 18F-fluorodeoxyglucose positron emission tomography (FDG-PET), amyloid-related biomarkers, and next-generation sequencing (NGS) testing.

**Results:**

Four patients, aged 25–45 years old, presented with a progressive UMN syndrome meeting clinical criteria of definite PLS. Cognitive symptoms and signs were mild or absent during the first year of the disease but appeared or progressed later in the disease course. Brain MRI showed microbleeds in two siblings, but iron-related hypointensities in the motor cortex were absent. Brain FDG-PET showed variable areas of hypometabolism, including the motor cortex and frontotemporal lobes. Amyloid deposition was confirmed with either cerebrospinal fluid (CSF) or imaging biomarkers. Two heterozygous likely pathogenic mutations in *PSEN1* (p.Pro88Leu and p.Leu166Pro) were found in the NGS testing.

**Conclusion:**

Clinically defined PLS is a syndrome encompassing different neurodegenerative diseases. The NGS testing should be part of the diagnostic workup in patients with PLS, at least in those with red flags, such as early-onset, cognitive impairment, and/or family history of neurodegenerative diseases.

## Introduction

Primary lateral sclerosis (PLS) is a rare neurodegenerative disorder characterized by a progressive upper motor neuron (UMN) impairment. Very few postmortem PLS cases have been described, sharing the same pathological signature that most cases of amyotrophic lateral sclerosis (ALS), namely neuronal cytoplasmic aggregates of TDP-43 (Turner and Talbot, 2020). However, unlike ALS, PLS causes progressive UMN degeneration without clinical or neurophysiological evidence of lower motor neuron (LMN) impairment (Turner and Talbot, 2020). Recently, new criteria for the clinical diagnosis of PLS have been proposed (Turner et al., 2020). However, there is considerable clinical overlap between PLS and other neurodegenerative diseases, such as hereditary spastic paraplegia (HSP) or the globular glial tauopathies (Brugman et al., 2009; Vázquez-Costa et al., 2016; Forrest et al., 2021). Consequently, the debate as to whether clinically diagnosed PLS can be considered a diseased part of the ALS spectrum, or rather a syndrome encompassing different neurodegenerative diseases, is ongoing (Mitsumoto et al., 2015; Turner et al., 2020).

Mutations in presenilin 1 (*PSEN1*) are the most common cause of autosomal dominant Alzheimer’s disease (ADAD). So far, over 300 *PSEN1* mutations have been identified.^1^ Despite most carriers usually present with early amnestic symptoms, atypical cognitive, and non-cognitive symptoms have been described in patients with *PSEN1*, including frontotemporal dementia (FTD), early aphasia, visual agnosia, myoclonus and seizures, extrapyramidal features, and spasticity (Riudavets et al., 2013; Tang et al., 2016).

In this study, we report four patients of two pedigrees meeting clinical criteria of definite PLS (Turner et al., 2020) but harboring *PSEN1* mutations.

## Family 1

**FIGURE 1:**
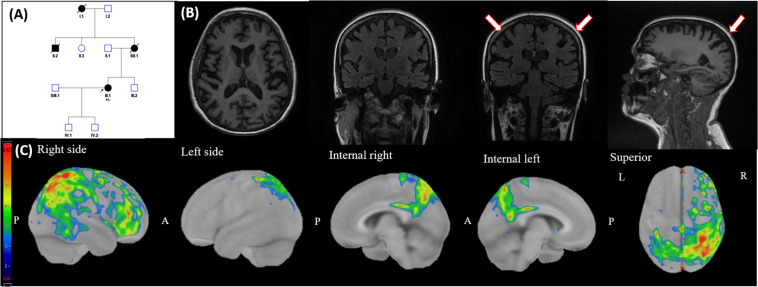
Family 1: pedigree, brain MRI, and 18F-fludeoxyglucose PET (FDG-PET). **(A)** Family pedigree. Dark symbols represent affected individuals, mother of proband died at age 64 after being diagnosed with early-onset dementia. Her maternal uncle was mentally retarded, and the grandmother of proband suffered an unspecified neurodegenerative disease. Arrow points to the proband. **(B)** Brain MRI showing widespread moderated cortical atrophy, strikingly in parietal lobes and precuneus (red arrows), but preserving hippocampus. **(C)** FDG-PET of the brain shows diffuse cortical hypometabolism in the right hemisphere; and hypometabolism in the precuneus, posterior cingulate, and paracentral gyrus of the left hemisphere. Images represent *z*-score deviations (warmer colors, more hypometabolism). P, posterior; A, anterior; L, left; R, right.

A 50-year-old Caucasian female was referred to the hospital after a 5-year history of dysarthria, followed 1 or 2 years later by dysphagia, gait imbalance, emotional lability, behavioral disturbances, and memory complaints. Her mother died at the age of 64 after being diagnosed with dementia 4 or 5 years before (Figure 1A).

At the time of her first assessment, 2 years after disease onset, she showed generalized UMN signs predominating in lower limbs and the Mini-Mental State Examination (MMSE) was 29/30. Brain and spinal cord MRI were unremarkable, and the patient was referred to the unit with the diagnosis of progressive pseudobulbar palsy.

Three years after disease onset, the patient showed at the physical exam dysarthria and saccadic intrusions into smooth-pursuit eye movements. Upper and lower limbs were spastic with generalized hyperreflexia and extension plantar response, and finger and foot tapping were slowed down. Sensory examination was unremarkable. Weakness, amyotrophy, and fasciculations were absent in all muscle groups.

The neuropsychological assessment revealed a mild-to-moderate cognitive impairment (MMSE: 20/30) characterized by bradypsychia, temporospatial disorientation, and recent memory deficit. Cognition was additionally examined with the Spanish version of the Edinburgh cognitive and behavioral ALS screen (ECAS) (Mora et al., 2018), showing a severe memory and language impairment and scoring 68/100 in ALS-specific tests (cut-off 53) and 13/36 in non-specific tests (cut-off 19). Behavioral impairment was evaluated after an interview with her relatives following the ECAS semistructured interview and the frontal system behavior questionnaire (FrSBe), meeting Rascovsky criteria (Rascovsky et al., 2011) for early apathy, disinhibition, and perseverative behavior.

Routine blood tests and serologies were unremarkable as was electromyography (EMG), which showed no signs of LMN impairment. Brain MRI showed unspecific cortical atrophy (Figure 1B), and iron-related hypointensity in SWI along the motor cortex (Vázquez-Costa et al., 2017) was absent (Figure 1B).

At this point, a tentative diagnosis of definite PLS and possible FTD was made according to current clinical criteria (Rascovsky et al., 2011; Turner et al., 2020).

The study was completed 3 months later with a positron emission tomography with 18F-fluorodeoxyglucose (FDG-PET), which revealed an asymmetric decrease of glucose uptake in frontoparietal areas, including the motor cortex (Figure 1C). Cerebrospinal fluid (CSF) analysis showed decreased levels of amyloid-β 42 (101 pg/ml; normal >725 pg/ml) and amyloid-β 42/40 ratio (0.031; normal >0.069) and increased levels of p-TAU_181_ (61 pg/ml; normal <56 pg/ml) and light-chain neurofilaments (NFL; 1,712 pg/ml; normal <830 pg/ml). A *C9orf72* expansion was excluded after a repeat-primed PCR. Next-generation sequencing (NGS) libraries were prepared using the SureSelect custom Constitutional Panel 17 Mb (Agilent Technologies, Santa Clara, CA, United States) according to the instructions of the manufacturer. This panel includes 5,227 genes involved in rare inherited disorders. These libraries were sequenced on a NextSeq 500 System using a NextSeq High Output V2 150 Cycles Reagent Kit (Illumina, San Diego, CA, United States). The resulting NGS data were analyzed with the Alissa Software tool (Agilent Technologies, Santa Clara, CA, United States). This analysis displayed a heterozygous rare variant (p.Pro88Leu) in the *PSEN1* gene, classified as likely pathogenic according to the American College of Medical Genetics and Genomics (ACMG) guidelines (Richards et al., 2015), whereas no other pathogenic or likely pathogenic mutations were found. After these results, the patient was diagnosed with ADAD.

## Family 2

**FIGURE 2:**
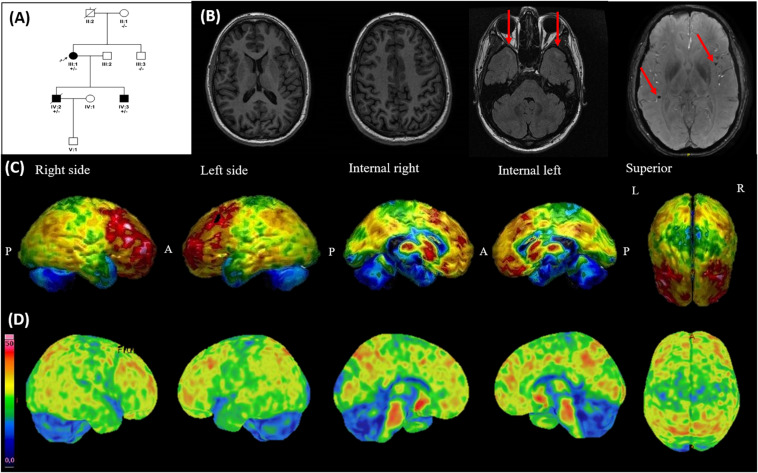
Family 2 pedigree, brain MRI, 18F-fludeoxyglucose PET (FDG-PET), and 18F-flutemetamol PET-TC. **(A)** Family pedigree: parents of the proband were unaffected, suggesting a *de novo* mutation. The arrow points to the proband. Dark symbols represent affected individuals. **(B)** Brain MRI of patient IV.2 at his 34 years old (6 years after disease onset). Left: two axial T1 images showing unspecific mild global cortical atrophy. Middle: axial FLAIR image, which reveals subcortical focal white-matter hyperintensities in the anterior temporal lobe (red arrows). Right: susceptibility-weighted (SW) image showing two microbleeds (red arrows). **(C)** FDG-PET of patient IV.3 shows bilateral hypometabolism in anterior temporal lobes, pre- and post-central gyri, and cerebellum. Colder colors represent lower glucose uptake. P, posterior; A, anterior; L, left; R, right. **(D)** 18F-flutemetamol PET of patient IV.3 shows diffusely increased amyloid binding in parietal and frontal lobes, sparing anterior temporal lobes, pre- and post-central gyri, and cerebellum. Colder colors represent lower amyloid binding.

A 37-year-an old Caucasian woman came to the clinic after a 1-year history of clumsiness and weakness in lower limbs, followed a few months later by clumsiness in upper limbs and dysarthria. Her personal and family history were unremarkable (Figure 2A). At that time, the neurologic examination showed generalized UMN signs, and MRI and EMG were normal. She was diagnosed with PLS. Some years later, memory complaints emerged, and the patient was finally institutionalized in an akinetic mutism state 10 years after the disease onset. Gastrostomy was placed 5 years after the institutionalization, but neither LMN signs nor respiratory insufficiency emerged.

A few years later, her two sons came consecutively to the clinic complaining of asymmetric clumsiness beginning in one lower limb at the age of 25 and 28 years old, respectively. This was followed by contralateral involvement short after, and clumsiness in upper limbs and dysarthria within 2–8 years from disease onset. Neither the patients nor their relatives complained of cognitive or behavioral symptoms. Their personal history was unremarkable, and both had received vocational training (as a mechanic and electrician) but were unemployed. The neurologic examination showed dysarthria and widespread UMN signs in bulbar, cervical, and lumbar regions, without LMN signs. The first neuropsychological exam showed normal MMSE in both patients (30), but borderline intelligence quotients (IQs) in the Wechsler adult intelligence test (WAIS-IV), including borderline values in verbal comprehension and processing speed indexes and normal working memory and perceptual reasoning indexes. The disease progressed toward anarthria and tetraparesis with pronounced spasticity, predominantly in lower limbs, and a baclofen pump was placed in one patient. No apparent progressive cognitive impairment appeared throughout the disease course, though the motor impairment precluded formal neuropsychological evaluation. However, they presented mild behavioral changes, mainly irritability and emotional lability, which were attributed to the motor impairment. The older patient died 14 years after the disease onset, probably due to dysphagia-related complications, as he declined gastrostomy placement.

In all three patients, routine blood tests and serologies were unremarkable, and neither clinical nor neurophysiological signs of LMN impairment were evident during the disease course. In both siblings, brain MRI showed mild unspecific cortical atrophy (Figure 2B). Scarcely scattered microbleeds together with focal white matter hyperintensities in the anterior temporal lobe were also visible in SWI and FLAIR, respectively (Figure 2B), but iron-related hypointensity in SWI along the motor cortex was absent. Brain FDG-PET of one sibling showed bilateral hypometabolism in anterior temporal lobes, pre- and post-central gyri, and cerebellum (Figure 2C).

Based on these findings a tentative diagnosis of definite PLS was made according to current criteria (Turner et al., 2020).

In all three patients, whole exome sequencing (WES) was performed after excluding a *C9orf72* expansion. WES was done using SureSelect Human All Exon v5 (Agilent) as capture system, paired in reads with 101 nucleotides and ran in an Illumina HiSeq2000 sequencing platform (Sistemas Genómicos). For the bioinformatics analysis, the patient was admitted to the Undiagnosed Rare Disease Program of CIBERER (ENoD). The p.Leu166Pro mutation, classified as likely pathogenic according to the ACMG guidelines (Richards et al., 2015), was found in all three patients and was absent in healthy relatives, including the mother of the proband (Figure 2A). No other pathogenic or likely pathogenic mutations were found in this analysis.

Following this, the youngest sibling underwent an 18F-flutemetamol PET, which revealed diffuse increased amyloid binding in parietal and frontal lobes, but sparing the hypometabolic areas in the FDG-PET (Figure 2D).

According to these results, patients were diagnosed with ADAD.

## Discussion

We describe four patients of two different pedigrees meeting clinical criteria for definite PLS, caused by two mutations in *PSEN1* (p.Pro88Leu and p.Leu166Pro).

Mutations in *PSEN1* are the most common cause of ADAD, and over 300 mutations have been described, some of them presenting with atypical phenotypes (Tang et al., 2016). The p.Pro88Leu and p.Leu166Pro mutations have been scarcely described before, but there is both preclinical and clinical evidence of their pathogenicity (Moehlmann et al., 2002; Lyoo et al., 2016; Liu et al., 2017) and are considered as likely pathogenic according to the current ACMG guidelines (Richards et al., 2015). Moreover, both have been related to young-onset AD and atypical features, such as spasticity, seizures, ataxia, and parkinsonism. However, typically, short-term memory impairment appeared before motor symptoms (Moehlmann et al., 2002; Lyoo et al., 2016; Liu et al., 2017). Conversely, in our patients, both motor and behavioral symptoms and signs preceded and predominated over cognitive symptoms, initially leading to misdiagnosis. Importantly, in both families, mutations in other ALS-causing genes were ruled out.

In the patient harboring the p.Pro88Leu mutation, the cognitive and behavioral symptoms progressed to cause dementia approximately 5 years after symptoms onset. Interestingly, while cognitive impairment predominantly affected language and memory, she also showed severe behavioral impairment, meeting the criteria for possible FTD. Accordingly, the FDG-PET not only revealed hypometabolism in brain areas typically impaired in AD (such as precuneus and posterior cingulate) but also affecting the right frontal lobe (Figure 1C). Interestingly, other PSEN1 mutations have been associated with both clinical and neuropathological (including Pick bodies) features of FTD, sometimes combined with typical AD features (Riudavets et al., 2013). In this case, CSF biomarkers oriented toward a diagnosis of AD, which was confirmed with the results of the gene panel.

More remarkably, in the patients harboring the p.Leu166Pro mutation, mild cognitive and behavioral symptoms appeared only several years after the motor symptoms onset and were masked by the motor symptoms. Although the father of proband could not be studied, the clinical and molecular information of the family (Figure 2A) suggests that a *de novo* mutation occurred in the proband. This is not surprising, since *de novo* mutations have been described in the same residue previously (Lyoo et al., 2016). Intriguingly, the FDG-PET in one of the patients showed hypometabolism in both anterior temporal lobes, pre- and post-central gyri, and cerebellum, a pattern neither suggestive of AD nor PLS (Van Laere et al., 2014). However, the disease progression toward akinetic mutism in the proband suggests that severe dementia might be a late feature in these patients. Moreover, the finding of microbleeds on brain MRI suggests the presence of amyloid angiopathy. Strikingly, the 18F-flutemetamol PET showed widespread amyloid deposition in the brain, except in the hypometabolic areas (Figure 2D), suggesting that amyloid was not responsible for the symptoms of patients. This is in agreement with a previous work, which suggested that Tau deposition in the primary motor cortex was responsible for the UMN signs in a patient harboring the same mutation (Lyoo et al., 2016). Moreover, previous studies suggest that non-amnestic symptoms are related to neurofibrillary tangles rather than to amyloid β plaques (Tang et al., 2016).

Interestingly, patients with globular glial tauopathy, including those harboring MAPT mutations, can also present as a UMN syndrome and be diagnosed with PLS (Erro et al., 2019; Forrest et al., 2021). All this suggests that some tauopathies can show a special tropism for UMN.

Other *PSEN1* mutations (p.Leu381Phe, p.Leu381Val, and p.Ala431Glu) have been recently described in patients clinically diagnosed with PLS (Jazi et al., 2019; Lewis et al., 2020). These and our mutations, all involve transmembrane domains, but this is not surprising, since most mutations are located in these domains. Moreover, other mutations in these same codons may associate with atypical symptoms but usually start with memory complaints.^2^ Furthermore, although some series have suggested that PSEN1 mutations after codon 200 are more likely to present spasticity than those before codon 200, this is not supported by the data nor by other series (Tang et al., 2016). Thus, the possibility of a genotype-phenotype correlation warrants further studies.

Primary lateral sclerosis is a mainly sporadic clinical syndrome consisting of a progressive UMN impairment frequently starting as spastic paraplegia and spreading rostrally to affect the cervical and bulbar region, usually within the first 10 years of the disease (Turner and Talbot, 2020). Some of these patients will develop LMN impairment during the disease course, and most pathological descriptions have shown cytoplasmic aggregates of TDP-43 in motor neurons (Pringle et al., 1992; Turner and Talbot, 2020). Consequently, PLS was proposed to be a disease of the ALS spectrum (Turner and Talbot, 2020; Turner et al., 2020). However, since there is currently no confirmatory diagnostic biomarker, the diagnosis remains clinical, based on a compatible clinical picture and disease course, together with the exclusion of mimics and other neurodegenerative disorders (Turner and Talbot, 2020; Turner et al., 2020).

Nevertheless, clinically diagnosed PLS and sporadic HSP shows a striking clinical and genetic overlap (Brugman et al., 2009; Mitsumoto et al., 2015; Vázquez-Costa et al., 2016; Yang et al., 2016). Consequently, many patients can be diagnosed either with PLS or with sporadic HSP, using current criteria (Vázquez-Costa et al., 2016). Furthermore, this clinical and genetic overlap includes other neurodegenerative diseases, such as the globular glial tauopathies (Erro et al., 2019; Forrest et al., 2021), atypical parkinsonisms (Mitsumoto et al., 2015), and, now, ADAD (Jazi et al., 2019; Lewis et al., 2020).

Although the patients met clinical criteria of definite PLS (Turner et al., 2020), the early onset, the presence of cognitive impairment, the family history of neurodegenerative diseases, and atypical findings in the FDG-PET and/or MRI were red flags that led us to expand the diagnostic workup. Therefore, in PLS cases with these atypical features, the use of amyloid-specific biomarkers should be considered. Moreover, since *de novo* or autosomal recessive mutations can cause a PLS-like syndrome, the screening of genes involved in neurodegenerative diseases should probably be part of the diagnostic workup of patients with PLS (Yang et al., 2016), even in the absence of family history. With the advances in genetics, it is foreseeable that new genes will join the list of PLS causes. Thus, and until TDP-43-specific biomarkers are available, PLS should remain considered a syndrome, encompassing different neurodegenerative diseases.

## Data Availability Statement

The datasets presented in this article are not readily available due to ethical restrictions. Requests to access the datasets should be directed to JV-C, juan.vazquez.neuro@gmail.com.

## Ethics Statement

The studies involving human participants were reviewed and approved by the Ethics Committee for Biomedical Research of the La Fe Hospital (2017/0653). The patients/participants and their relatives provided their written informed consent to participate in this study for genetic analysis and the use of their anonymized data for research purposes. Written informed consent was obtained from the individual(s) for the publication of any potentially identifiable images or data included in this article.

## Members of the ENoD Consortium

Beatriz Morte, Rosario Carmona, Javier Perez-Florido, Virginia Aquino, Francisco Ortuño, Daniel Lopez-Lopez, Gerrit Bostelmann, Joaquin Dopazo, and Luis Alberto Pérez-Jurado are part of the Undiagnosed Rare Disease Program from the Centre for Biomedical Research on Rare Diseases (ENoD-CIBERER).

## Author Contributions

JF-VC designed the study, participated in clinical data acquisition and interpretation, and wrote and edited the manuscript. MP-M participated in clinical data acquisition and interpretation, and edited the manuscript. MM-M, MM, PS-N, TJ, JS, ENoD Consortium and JP-T participated in clinical or technical data acquisition and interpretation, and critically revised the manuscript. TS designed the study and critically revised the manuscript. All authors have approved the submitted version of the manuscript.

## Conflict of Interest

JV-C received personal fees from Roche. The remaining authors declare that the research was conducted in the absence of any commercial or financial relationships that could be construed as a potential conflict of interest.

## Publisher’s Note

All claims expressed in this article are solely those of the authors and do not necessarily represent those of their affiliated organizations, or those of the publisher, the editors and the reviewers. Any product that may be evaluated in this article, or claim that may be made by its manufacturer, is not guaranteed or endorsed by the publisher.

## Footnotes

1
https://www.alzforum.org/mutations

2
https://www.alzforum.org/mutations/psen-1
